# The Emerging Neurobiology of Bipolar Disorder

**DOI:** 10.1016/j.tins.2017.10.006

**Published:** 2018-01

**Authors:** Paul J. Harrison, John R. Geddes, Elizabeth M. Tunbridge

**Affiliations:** 1 Department of Psychiatry, University of Oxford, Warneford Hospital, Oxford, OX3 7JX, UK; 2 Oxford Health NHS Foundation Trust, Warneford Hospital, Oxford, OX3 7JX, UK

**Keywords:** digital phenotyping, genomics, L-type calcium channels, mood disorder, mood instability, therapeutics

## Abstract

Bipolar disorder (BD) is a leading cause of global disability. Its biological basis is unknown, and its treatment unsatisfactory. Here, we review two recent areas of progress. First, the discovery of risk genes and their implications, with a focus on voltage-gated calcium channels as part of the disease process and as a drug target. Second, facilitated by new technologies, it is increasingly apparent that the bipolar phenotype is more complex and nuanced than simply one of recurring manic and depressive episodes. One such feature is persistent mood instability, and efforts are underway to understand its mechanisms and its therapeutic potential. BD illustrates how psychiatry is being transformed by contemporary neuroscience, genomics, and digital approaches.

## Trends

BD is highly heritable and mostly attributable to common variants of small effect. Several risk genes and gene networks have been identified.

Calcium signalling is prominent among the genetic risk pathways, and currently appears to have the greatest therapeutic traction.

Digital technologies and sophisticated mathematical and computational analyses are being used to quantify and understand BD.

These new methods reflect, and are promoting, reconceptualisation of BD as a chronic instability of mood and neural circuitry.

Stem cells are becoming an integral part of the approaches to understanding BD and its pharmacotherapy.

New experimental medicine models are being applied to identify and rapidly test potential mood-stabilising treatments.

## Bipolar Disorder and the New Psychiatry

Psychiatry still relies largely on 19th-Century diagnostic categories. These are based on clusters of symptoms rather than biological markers, and are treated with drugs discovered serendipitously several decades ago. BD typifies this unsatisfactory state of affairs. Although its name has changed [it was formerly known as **manic depression** (see Glossary)], its cardinal features, and how it is assessed and treated (Box 1) have barely altered. An important reason for this stagnation has been the lack of any real traction on its causes and underlying biology, beyond its well-established high heritability [1]. Although there is evidence for altered structural and functional brain connectivity 2, 3, 4, and changes in markers of oxidative stress [5], mitochondrial function [6], inflammation [7], circadian rhythms [8], and dopamine [9], it remains difficult to integrate these diverse findings, and to disentangle causative changes from those that are secondary to the disorder and its treatment.

Box 1 **Bipolar Disorder: A Clinical Primer**

The classic picture of BD is like a modified sine wave, with mood fluctuating between episodes of mood elevation (mania) and depression, interspersed with periods of euthymia. The number of episodes in each mood phase, and their duration, varies markedly between individuals, but for most patients the depressive episodes are more prolonged and are responsible for much of the morbidity of the disorder.

The depressive episodes of BD are broadly similar in nature and severity to those of ‘ordinary’ depression. A manic episode includes not only significant elevation of mood, but also related changes in behaviour, such as a reduced need for sleep, increased energy, grandiose thoughts and beliefs, rapid speech, increased libido, and reckless behaviour (e.g., spending excessively). In severe episodes, psychotic symptoms (delusions and hallucinations) may also be present; for example, the person may believe, or hear voices telling them, that they have superpowers (and they may then act accordingly). ‘Hypomania’ refers to a milder and less prolonged form of mania. The exact criteria depend upon the classificatory system used (**ICD-10** or **DSM-5**); the latter subdivides BD into bipolar I and bipolar II. Although not part of the diagnostic criteria, cognitive impairment is a notable aspect of BD; it is present at first episode and persists during euthymia. Attention, processing speed, and verbal learning and fluency are the domains most affected. At least half of patients with BD also have an anxiety disorder or substance use disorder. Patients are typically diagnosed during their 20s, following a long prodrome, and actual onset is often in adolescence. The lifetime prevalence of BD is approximately 1%, rising to 4% if a broader definition of **bipolar spectrum disorder** is used. The major risk factors are genetic (see the main text), but environmental factors, including childhood adversity, also have a role. Of patients with BD, 10% die by suicide and this, coupled with an excess mortality from natural causes, shortens average life expectancy by approximately 15 years.

The mainstay of BD treatment is pharmacological, with lithium salts or the antiepileptic drug sodium valproate used for prophylaxis. Antipsychotics, antidepressants, and antiepileptic drugs are given to treat the mood episodes. Psychoeducation and psychological treatments also have an important supporting role. All current treatments have limited efficacy, and the drugs can have serious adverse effects.

For an introduction to clinical aspects of BD, see 10, 95.

Alt-text: Box 1

The situation is belatedly improving. While optimism must be tempered by appreciation of the many complexities, there are realistic prospects for a transformation in our understanding of BD and how it is diagnosed and treated. Here, we highlight two areas of current interest: the discovery of the first BD risk genes and their implications, and the application of novel technologies with the potential to refine, or redefine, the BD phenotype. These developments exemplify how genomics, neuroscience, and digital technologies are heralding a new era for psychiatry. For broader reviews of BD, see 10, 11.

## The Genomics of Bipolar Disorder

A child of an affected parent has about a tenfold increased risk of developing BD, and twin studies estimate a heritability of 0.7–0.8 [1]. There is no evidence for Mendelian inheritance or for genes of major effect. Instead, as with most psychiatric disorders, there are multiple susceptibility loci, each of small effect, which genome-wide association studies (GWAS) are beginning to identify. Several GWAS, and meta-analyses thereof, have been carried out since 2007; Table 1 lists the loci and implicated genes that have emerged to date. The combined sample sizes remain small by GWAS standards, and more loci remain to be identified; indeed, the forthcoming Psychiatric Genomics Consortium analysis, comprising over 20 000 BD cases and 30 000 controls, identifies 19 significant loci, including 12 novel ones. Initial exome and genome sequencing data suggest that rare deleterious variants also have a role in some BD cases, but their identity and overall contribution to the disorder remain unclear 12, 13, 14. Within BD, there is modest clinicogenetic heterogeneity, for example, based on the predominant symptoms, or between **bipolar I** and **bipolar II** subtypes [15]. However, there is little evidence for BD-specific genes; joint GWAS analyses show substantial commonalities in risk loci for BD and schizophrenia [16], as well as significant overlap with other major psychiatric disorders [17] and with intermediate phenotypes, including circadian traits 18, 19. One distinction between schizophrenia and BD is that copy number variation is much less prominent in the latter [20].

**Table 1:** GWAS Hits for BD^a^

Locus	Implicated gene(s)
2q11.2	*LMAN2L*
2q32.1	*ZNF804A*
3p22.2	*TRANK1* (*LBA1*)
5p15.31	*ADCY2*
6q16.1	*MIR2113*, *POU3F2* (*OTF7*)
6q25.2	*SYNE1*
7p22.3	*MAD1L1*
9p21.3	Intergenic
10q21.2	*ANK3*
11q14.1	*TENM4* (*ODZ4*)
12p13.3	*CACNA1C*
12q13.1	*DDN*
17q12	*ERBB2*

a Data from 98, 117.

Although the genomics of BD are in their infancy, efforts have begun to understand the biological basis for the associations identified to date. Interest has centred on two genes (*CACNA1C* and *ANK3*) because of what was already known of their functions. *CACNA1C* is discussed in detail below. *ANK3* encodes ankyrin G, which couples axonal voltage-gated sodium channels to the cytoskeleton and also has roles in dendrites and glia; another risk gene, *TRANK1*, contains multiple ankyrin repeat domains, suggesting some shared functions. Complementing the focus on specific risk genes, the first attempts have been made to identify the pathways that they influence. Using data from four of the GWAS, Nurnberger *et al.* [21] reported six pathways that showed replicable association with BD, involving glutamate and calcium signalling, second messengers, and hormones. Together, these findings support the possibility that BD is, at least in part, an ion channelopathy [22], in which aberrant calcium signalling is important [23].

## Calcium Signalling in Bipolar Disorder: Linking Genetics, Pathophysiology, and Therapeutics

Calcium dysregulation has long been implicated in BD, based primarily on *ex vivo* studies in cells taken from patients and controls. The findings are disparate, but on balance indicate that measures of intracellular calcium signalling are increased in BD, especially after stimulation (reviewed in 23, 24). The abnormalities appear largely independent of current mood state (i.e., they are trait rather than state related). Moreover, they are attenuated by lithium, which is used in the treatment of the disorder (Box 1). Despite the many uncertainties, these findings led to L-type voltage-gated calcium channel (VGCC) antagonists, with existing indications in angina and hypertension, being evaluated for the treatment of BD [25]. Antiepileptic drugs, such as pregabalin, which act via VGCC α_2_δ subunits (Box 2) have also been tested [26], and lamotrigine, another antiepileptic drug that may block calcium channels, among its various actions [27], is an effective treatment for bipolar depression [28].

**Figure I:**
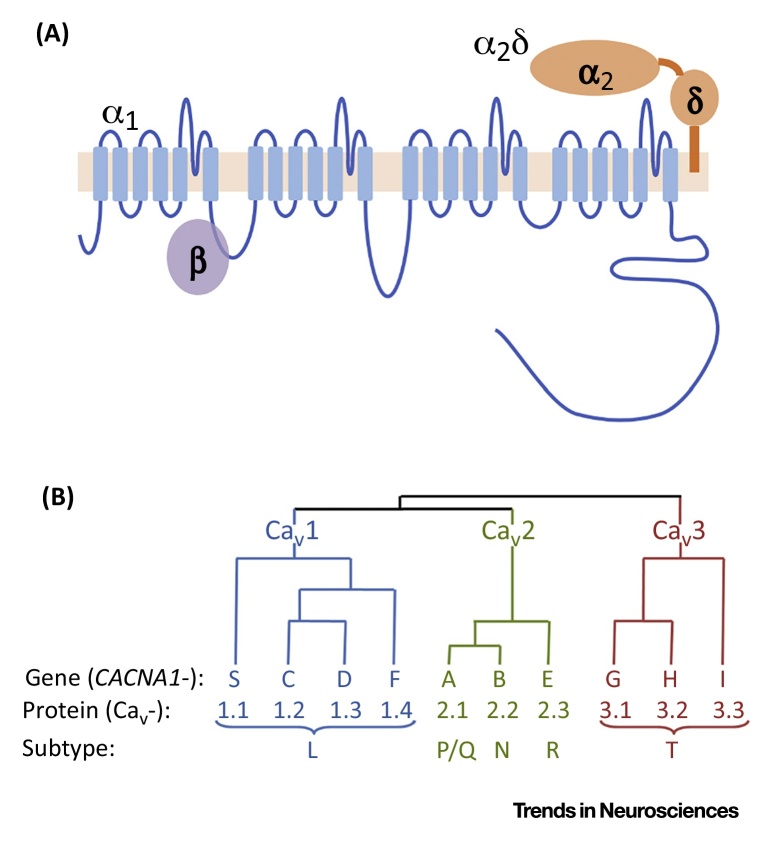
The Voltage-Gated Calcium Channel (VGCC) Family of Proteins. (A) Structure of VGCCs, showing the transmembrane topology of the α1 subunit, its long intracellular C terminus and interactions with accessory (β and α_2_δ) subunits. (B) Dendrogram and nomenclature of the VGCC family.

Box 2 **VGCC Genes, Their Isoforms, and Relevance in Bipolar Disorder**

Identifying the specific VGCCs most relevant for BD is a significant challenge, because their genes give rise to a vast diversity of functional channels (named Ca_v_ channels) 96, 97. VGCCs comprise multiple subunits, each encoded by one of a subfamily of separate genes. The properties of the ten distinct α1 subunits (encoded by the *CACNA1*- gene family) depend on the accessory subunits to which it is bound (Figure I). The main accessory subunits are the β (encoded by *CACNB1*–*4*) and α_2_δ (encoded by *CACNA2D1*–*4*) subunits, which are obligate in most cases [98]. Current VGCC antagonists block the L-type channels; the anticonvulsant/analgesic drugs pregabalin and gabapentin are α_2_δ ligands [30].

Further channel diversity arises because each gene gives rise to multiple isoforms. The human *CACNA1C* mRNA has at least 50 exons and over 40 predicted isoforms (arising from transcriptional and splicing mechanisms). *CACNA1C* splicing gives rise to channel isoforms that are differentially expressed in brain compared with heart, and which differ in their biophysical properties, including voltage-gating characteristics [97]. Another feature affected by splicing is the isoform sensitivity to existing VGCC antagonists [98]. This suggests that it might be possible to selectively target splice variants that mediate disease risk and/or are preferentially expressed in the brain, compared with peripheral tissues (particularly the cardiovascular system, where VGCCs are also abundant), thereby maximising their therapeutic potential and tolerability in BD [25].

Given these considerations, defining the repertoire of VGCCs present in different human tissues is important, as is identifying which ones are impacted by the BD-associated risk variants or by BD itself. However, information on the transcript diversity of human VGCC subunits is sparse, particularly in brain. Furthermore, because VGCC subunit genes are large (full-length *CACNA1C* mRNA, for instance, is over 10 kb long), the transcript structure of most isoforms remains unclear. Characterising the profile of full-length VGCCs isoforms in the human brain, compared with other tissues, and assessing which are altered in association with genetic risk for BD, are critical first steps in translating the VGCC genomic findings into pathophysiological insights and novel treatment targets. The availability of large, high-quality human postmortem brain series and technological advances in the field of RNA sequencing make this goal achievable.

Alt-text: Box 2

The results of the recent genomic studies strongly suggest that the involvement of calcium signalling in BD is at least partly causal [29], and have rekindled attempts to explain more precisely the nature of the alterations, not least because this may provide clues to more-effective and tolerable drug strategies to normalise them [30]. However, the discovery of genetic variants is only the first step, and provides many more questions than answers. Calcium signalling offers an informative exemplar to highlight the opportunities and complexities associated with moving from psychiatric genomic discoveries to pathophysiological insights and therapeutic advances 31, 32.

Genomic data provide a starting point to identify the molecules involved in the core ‘calcium pathophysiology’ of BD. They focus attention on the VGCCs, especially of the L-type, and their accessory subunits (encoded by the *CACNx* genes; Box 2). As indicated above, the best evidence is for *CACNA1C* (encoding the α1 subunit of Ca_v_ 1.2), but pathway analysis also suggests a role for *CACNA1D* and *CACNB3* 29, 33, and other VGCC genes are implicated in BD by rare variant studies [13]. Of note, apart from BD, *CACNA1C* is associated with schizophrenia [34] and major depression [35], and *CACNB2* confers susceptibility to multiple psychiatric disorders [17]. Involvement of VGCC genes has also been reported in large-scale genomic studies of BD-relevant phenotypes, such as working memory performance and the associated patterns of brain activation [36], as well as in general cognitive functioning [37]. There are also smaller candidate gene studies that suggest effects of *CACNA1C* genetic variation on brain imaging phenotypes 38, 39 and on cognitive domains, such as reward responsiveness [40].

Identification of the molecular basis for disease associations is a key step in understanding the mechanisms linking VGCC genes with BD. The VGCC loci revealed by GWAS are noncoding, and while large-scale exome studies may identify rare variants that disrupt the coding sequence of VGCCs, it is unlikely that the BD-associated GWAS loci tag as-yet-unidentified, disease-causing mutations. Instead, they probably act by influencing aspects of gene expression, including methylation [41], alternative promoter usage, and RNA splicing [42]. In the case of *CACNA1C*, the index risk polymorphism for BD (rs1006737) is located in the third intron. On balance, the available data indicate that the risk allele is associated with enhanced expression and activity of the gene product 43, 44, but there are conflicting findings 45, 46, precluding firm conclusions. Some of the variability in these results may be due to differences in the effect of rs1006737 on *CACNA1C* expression between brain regions. Inconsistencies may also result from the risk single nucleotide polymorphism (SNP) differentially altering the abundance of particular splice variants, as has been observed for other BD-relevant genes, including *ANK3* [47] and *ZNF804A* [48]. In the case of *ANK3*, there is evidence that the shift in isoform ratios has functional consequences for neuronal physiology [49]. Efforts to identify and understand whether altered splicing is also relevant for *CACNA1C* and other VGCCs are hampered by the limited information about their isoform profile in the human brain (Box 2). This information is critical since splicing patterns are poorly conserved and the brain shows one of the greatest diversities of alternative splicing [50], meaning that the current data, which pertain to other species and tissues, are insufficient.

While the identification of specific VGCC subunits and isoforms that mediate BD risk is important, better understanding of the underlying biology is also crucial. This requires the use of appropriate cellular and animal model systems, as well as *in vivo* approaches in humans. For cellular analyses, in addition to standard cell lines, which are useful for studying the function of individual genes, induced pluripotent stem cells (iPSCs) may prove valuable. Indeed, iPSC-derived neurons have already provided intriguing data to support the presence of cellular BD-related phenotypes, including alterations in calcium signalling (Box 3).

Box 3 **Stem Cells and the Calcium Pathophysiology of Bipolar Disorder**

The potential of iPSCs for studying cellular phenotypes of psychiatric disorders such as BD is considerable, and the approach has already been exploited in several studies. These have been of three designs: comparison of BD with healthy patients; contrasting lithium-responsive versus lithium-unresponsive BD cases; or evaluating the effect of BD risk genotypes (reviewed in [99]).

Mertens *et al*. [100] generated dentate gyrus-like neurons and showed that cells from patients with BD were hyperexcitable, with differences in several electrophysiological and transcriptional parameters; moreover, the excitability was normalised by lithium, but only in cells derived from patients who had responded clinically to the drug. Notably, these findings were largely replicated in a separate cohort and using a different methodology [101]. Taking a genetic strategy, Yoshimizu *et al.* [44] made induced neurons from 24 people, genotyped for the *CACNA1C* rs1006737 polymorphism, and showed that *CACNA1C* gene expression and calcium current density were greater in risk allele homozygotes compared with nonrisk carriers.

3D differentiation approaches are now being used to produce ‘brain spheroids’. Although no studies of this kind have yet been reported using iPSCs from patients with BD, Birey *et al.* [102] reported that, intriguingly, spheroids made with iPSCs from patients with Timothy syndrome (caused by a gain-of-function coding *CACNA1C* mutation) exhibited interneuron migratory abnormalities that could be normalised by a VGCC antagonist. The finding draws attention to a possible role for early developmental events and specific interneuron populations in mediating the role of calcium signalling in the pathogenesis of BD. Studies using spheroids in BD may be valuable in helping identify circuit-level phenotypes, while CRISPR techniques to selectively manipulate disease-relevant VGCC isoforms and variants could aid the identification of the key molecular mechanisms.

These findings, and others (e.g., [103]), illustrate how iPSCs are providing new clues regarding the neuronal phenotypes of BD, and support an involvement of calcium signalling in these processes. The results encourage further efforts to extend and scale up the work [104].

Alt-text: Box 3

The molecular findings can also help guide the development of rodent models overexpressing or lacking specific VGCC genes 51, 52, 53 or splice variants thereof [54], in which their functional impact can be studied in the intact animal. For example, embryonic deletion of Cacna1c from forebrain glutamatergic neurons in mice produced BD-relevant behavioural and cognitive effects and an increased susceptibility to stress, whereas the same deletion during adulthood caused a lesser and, in some instances, opposite phenotype [51]. This effect of developmental stage on the phenotype is intriguing, given the characteristic early adulthood age of BD onset, and its childhood antecedents (Box 1). In a separate study, the phenotype of Cacna1c-deficient mice could be rescued using a small-molecule inhibitor of the translation initiation factor eIF2α [52], giving clues as to the possible intervening biochemical mechanisms.

In parallel with these various genetically driven approaches, pharmacological investigations of VGCCs in humans are possible because of the existing L-type VGCC antagonists, which can be used as experimental tools and for proof-of-principle studies. Their availability is a distinct advantage compared with most other genetically supported therapeutic targets in BD, for which no such drugs are available. To date, the clinical trial data linking L-type VGCC blockade with therapeutic outcome in BD (and, indeed, their psychiatric effects more generally) are wholly inconclusive [25]. Hence, a priority is to investigate in detail the impact of brain-penetrant VGCC blockers on BD-relevant phenotypes, including detailed measures of mood, cognition, sleep, and brain activity [55], and with the incorporation of genotype as a factor [56]. The potential psychiatric effects of VGCC antagonists can also be assessed using routinely collected clinical data; for example, a study of electronic medical records in Scotland reported higher hospital admission rates for depression in patients given VGCCs compared with those prescribed other antihypertensives [57].

**Figure 1:**
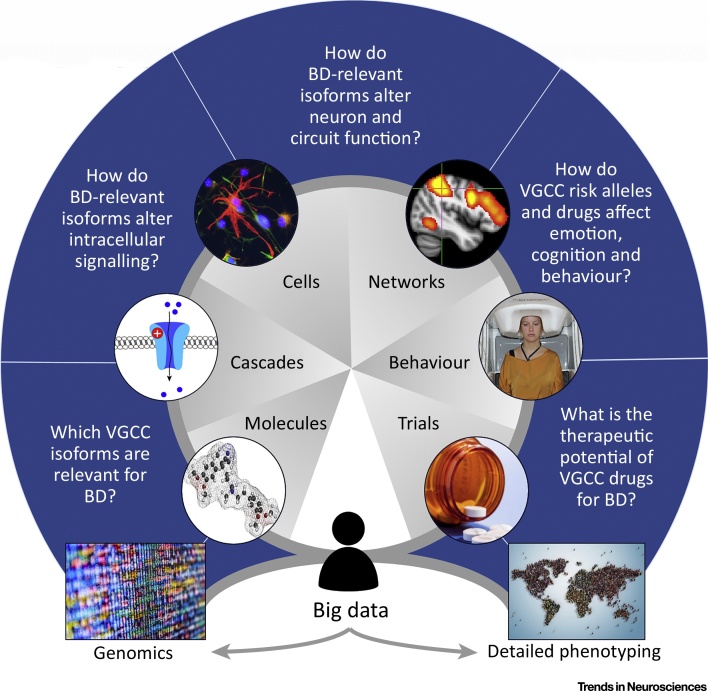
Key Figure: Genomics, Neuroscience, and Treatment Innovation in Bipolar Disorder (BD)

In summary, a range of methods are needed to make the most of the genomic discoveries in BD, to understand their molecular mechanisms and implications for cellular and systems functioning, and to evaluate their therapeutic potential. The conceptual approach is illustrated in Figure 1 (Key Figure), which uses VGCCs as the exemplar.

## New Approaches to the Bipolar Phenotype

### Digital Psychiatry

Complementing the developments from genomics-driven discovery science, novel methods are being applied to characterise the BD phenotype, and thereby provide new perspectives on its key elements.

Conventional psychiatric assessments rely on cross-sectional, retrospective analysis of the pattern of symptoms over weeks or months. Being based on a patient’s or informant’s recall, this approach is subject to inherent biases and unreliability, especially when, as in BD, the key feature is not simply a symptom (mood), but its profile of variation over time. Several approaches have been used to try to improve the reliability and validity of BD assessments 58, 59. The main advances are emerging from developments in information technology, including the increasingly widespread use of connected and wearable devices: we are entering an era of ‘digital phenotyping’ in psychiatry [60], with BD, we would argue, at the forefront [61]. One early example is the True Colours platform, which allows patients to submit (by text, email, web, or app) their ratings of depression, mania, and other symptoms, in response to a weekly or daily prompt, resulting in a longitudinal and graphical representation of symptom course [62].

Importantly, smartphones and other devices allow the remote capture of not only contemporaneous self-reporting of symptoms, but also behavioural, cognitive and physiological measures, such as heart rate, activity, geolocation, speech, and environmental interactions 63, 64, 65, 66, 67, 68. In turn, digital methods can facilitate a change from entirely symptom-based characterisation of psychiatric disorders, such as BD, towards a more multimodal, biologically informed one. Thus, they raise the prospect of more-objective, data-driven diagnoses, and ultimately personalised predictions of illness and treatment response. However, this promise has yet to be realised; the initial hype about digital phenotyping, including its application to BD, has been tempered by increasing awareness of the significant technical, analytical, and other issues involved (Box 4).

Box 4 **Digital and Mathematical Approaches to Bipolar Disorder**

Collection of multidimensional data, as in the remote monitoring of symptoms, behaviours, and physiology, has considerable potential in the study and treatment of BD and other disorders, but also throws up several significant challenges 105, 106.

First, there are many technical and practical issues to overcome, ranging from ensuring the compatibility of data collection between operating systems and software versions, to maintaining compliance over long time periods. There are also privacy, acceptability, and engagement issues raised by the recording and storage of personal data [107].

Second, the resulting data sets are large and complex, and the extraction, analysis, and interpretation of information are not straightforward [108]. One key question to consider is the mathematical approach chosen for analysing the data. Options include linear and nonlinear time series methods 109, 110, 111 as well as more-flexible and advanced techniques, such as relaxation oscillator frameworks [112] and machine-learning methods [90]. Another mathematical technique of interest is rough paths theory [113]. By taking account of lead-lag relationships, rough paths can reduce time-stamped data sets from complex interacting nonlinear systems to their critical information content or ‘signature’. This transformation provides a structured sequence of low-dimensional summaries of the primary data that completely characterise their complexity but are amenable to linear analysis. This greatly facilitates efforts to use the data for classification and prediction. For example, a rough path signature, based on daily measures of mood instability, can differentiate BD from borderline personality disorder [114].

Progress in this developing field will need to be interdisciplinary, combining advanced mathematical analyses with digital phenotyping technologies that produce robust data and that are sufficiently acceptable, and rewarding, to patients to secure their long-term engagement 115, 116.

Alt-text: Box 4

### Persistent Mood Instability

Notwithstanding the many challenges, the longitudinal, long-term collection of data using digital approaches has already contributed to a renewed focus on the ‘real-world’ phenotype of BD. One example is the appreciation that many patients have chronic mood instability (also called **affective** lability), persisting during **euthymia**. This contrasts with the simplistic textbook description of BD as comprising periods of normal, stable mood in between the episodes of depression and mania (Box 1). Although this reality was already appreciated by experienced clinicians 69, 70, it is the use of digital methods that has led to a greater awareness of mood instability, which in turn has encouraged research to understand its origins and significance. Here, we briefly review some of these implications.

Mood (in)stability is a continuous variable, and is present to varying extents in healthy individuals 71, 72. Therefore, it is a useful phenotype for studies seeking to identify mechanisms underlying mood (dys)regulation *per se*, and a range of experimental approaches are being taken. For example, it can be viewed from a computational perspective, with models showing how mood instability interacts with reward sensitivity to alter performance [73]. One can also ask how mood instability relates to variation in neural activity across a range of temporal resolutions, using functional MRI [74] and magnetoencephalography [75]. In addition, mathematical analyses, such as those outlined in Box 4, can help explain the relationships between fluctuations in mood and other parameters, ranging from environmental exposures to behavioural variables 66, 67.

Mood instability is prominent not only in BD, but also in several other psychiatric disorders, such as attention deficit disorder, borderline personality disorder, and schizophrenia 76, 77. It is therefore a trans-diagnostic construct, compatible with the NIMH Research Domain Criteria project [78]. Thus, understanding the origins and mechanisms of mood instability, and the biological and behavioural sequelae of interventions which modify it – is therefore likely to have value beyond BD. Indeed, mood instability is a moderately heritable trait in the general population, and the first genome-wide significant loci have been reported [79]. Apart from studying the implicated molecules and pathways in their own right, it will be of interest to investigate the extent to which the risk loci for mood instability overlap with those for BD specifically, and other disorders involving mood instability. Moreover, the specific characteristics of mood instability (e.g., its frequency, amplitude, or impact on behaviour), while partially overlapping, may differ sufficiently between disorders [80] to help gain traction on the underlying neural mechanisms, providing further clues to the biological bases of these illnesses and leading to refinements in classification.

Finally, mood instability has potential immediate value as a therapeutic target. First, it may provide a way to screen novel treatments for BD more rapidly and cost-effectively than conventional clinical trials, in which treatment or prevention of depressive or manic episodes are the usual outcomes, requiring prolonged periods of observation. In this sense, early mood stabilisation could prove to predict therapeutic efficacy in the way that rapid effects on emotional processing predict subsequent therapeutic response in unipolar depression [81]. Moreover, the rapid effects of antidepressants on emotional processing are seen in euthymic subjects as well as in those with depression; similarly, new treatments for BD, such as novel VGCC-acting drugs, could be screened in patients who have unstable mood but who do not have a formal diagnosis. Second, stabilisation of mood may be beneficial in its own right, above and beyond simply preventing clinical episodes of depression and mania, because mood instability independently predicts poor functional recovery in BD, and worse outcomes of various kinds 76, 82, 83, 84. In addition, since mood instability often occurs before the onset of BD 85, 86, such treatments might have a preventative role.

In summary, the emergence of persistent mood instability as a construct illustrates how digital methods can highlight neglected or hard-to-characterise psychiatric phenomena, and stimulate a range of studies into their origins, mechanisms, and therapeutic implications. Comparable approaches are being taken to other components of BD, such as circadian dysregulation and reward sensitivity. Although the work is at an early stage, it is likely ultimately to alter the view of the core phenotype of BD, with a greater role for biological and quantitative data in BD diagnosis, prognosis, and management.

## Concluding Remarks

Our understanding of BD remains frustratingly limited. It continues to be a descriptive syndrome, since we lack sufficient knowledge to allow its characterisation or conceptualisation based on aetiology or mechanism. Certainly, many questions remain (see Outstanding Questions). However, there are reasons for optimism. First, the discovery of some of the BD risk genes has the potential to revolutionise our understanding of its pathogenesis and neurobiology. Second, the use of digital technologies and remote sensors, coupled with advanced analyses of the resulting data, is already allowing a more-quantitative, longitudinal approach to the BD phenotype. This raises the potential for better prediction of an individual’s clinical course, and also provides a more-sophisticated phenotype for behavioural and biological studies. In both of these domains, a common feature is the increasing importance of ‘big data’, whether in terms of the genomic studies, or the multidimensional data streams captured by digital devices. Third, although not discussed here, structural and functional brain imaging is helping to identify the key neural circuits of BD and may also have diagnostic and prognostic value 2, 3, 4, 87, 88, 89, 90.

The ultimate goal of these contemporary approaches is to allow a more scientifically informed, evidence-based approach to how BD is classified, measured, and treated [91]. Possible changes include redrawing or removing the diagnostic boundaries between BD and other disorders involving lability of mood, emotion, and behaviour; the inclusion of behavioural and physiological correlates of mood and mood instability (or other symptoms) into clinical practice; a focus on identifying interventions that can stabilise mood independent of the underlying diagnosis; and new, genetically informed treatments, such as those targeting VGCCs.

It is not always appreciated that BD causes a global health burden comparable with that of schizophrenia [92], yet it has attracted considerably less research funding and interest from policy makers [93]. We would argue that, for various reasons, BD currently has a greater potential for transformative advances. More generally, BD is a useful case study for illustrating how psychiatric disorders are belatedly embarking on the journey from being descriptive syndromes towards more neurobiologically grounded, quantitative, and digital phenotypes [94]. Despite the many difficulties this process entails, it is not unreasonable to hope that it will be successful, and accompanied by the development of more rational, effective, and personalised treatments.

Outstanding Questions What is the overall genetic architecture of BD? What are the main pathways impacted by the genes?

How will genetics alter the diagnosis of BD and its relationship to other disorders?

What are the causes and mechanisms of altered calcium signalling in BD?

What is the core neurobiology of BD, at systems and cellular levels?

Will genetic findings and novel phenotyping lead to improved therapies for BD?

## Glossary

**Affect(ive)**: essentially synonymous with mood. BD is sometimes known as ‘bipolar affective disorder’.
**Bipolar I disorder**: the ‘textbook’ form of the disorder, with full manic episodes.
**Bipolar II disorder**: a form of the disorder in which manic episodes are milder or briefer, and depression predominates.
**Bipolar spectrum disorder**: includes bipolar I and II, and other cases not meeting criteria for those disorders.
**Cyclothymia**: a personality type characterised by ups and downs of mood, but lacking the severity or functional impact of BD.
**DSM-5**: the fifth edition of the *Diagnostic and Statistical Manual of Mental Disorders.* Includes formal diagnostic criteria for BP. Published by the American Psychiatric Association in 2013 and widely used in research.
**Euthymia (or euthymic)**: normal mood, neither manic nor depressed.
**ICD-10**: the tenth edition of the *International Classification of Mental and Behavioural Disorders* was published by the World Health Organisation in 1992. ICD-10 describes the clinical features of BD but is rarely used in research since DSM-5 criteria are operationalised and more detailed. ICD-11 will be released in 2018.
**Manic depression (or manic depressive psychosis)**: an older term for BD.
**Mixed affective states**: when depressive and manic features are present simultaneously.
**Psychotic BD**: a severe form of BD, in which psychotic symptoms (delusions and hallucinations) occur during manic and/or depressive episodes.
**Rapid cycling**: occurrence of four or more episodes (or mania or depression) within a year.
